# Activity of ertapenem/zidebactam (WCK 6777) against problem Enterobacterales

**DOI:** 10.1093/jac/dkac280

**Published:** 2022-08-16

**Authors:** Shazad Mushtaq, Paolo Garello, Anna Vickers, Neil Woodford, David M Livermore

**Affiliations:** Antimicrobial Resistance and Healthcare Associated Infections Reference Unit, Reference Services Division, United Kingdom Health Security Agency, 61 Colindale Avenue, London NW9 5EQ, UK; Antimicrobial Resistance and Healthcare Associated Infections Reference Unit, Reference Services Division, United Kingdom Health Security Agency, 61 Colindale Avenue, London NW9 5EQ, UK; Antimicrobial Resistance and Healthcare Associated Infections Reference Unit, Reference Services Division, United Kingdom Health Security Agency, 61 Colindale Avenue, London NW9 5EQ, UK; Antimicrobial Resistance and Healthcare Associated Infections Reference Unit, Reference Services Division, United Kingdom Health Security Agency, 61 Colindale Avenue, London NW9 5EQ, UK; Antimicrobial Resistance and Healthcare Associated Infections Reference Unit, Reference Services Division, United Kingdom Health Security Agency, 61 Colindale Avenue, London NW9 5EQ, UK; Norwich Medical School, University of East Anglia, Norwich, Norfolk NR4 7TJ, UK

## Abstract

**Background:**

Secondary healthcare will remain pressured for some years, both because SARS-CoV-2 will circulate as a nosocomial pathogen, and owing to backlogs of patients awaiting delayed elective procedures. These stresses will drive the use of Outpatient Parenteral Antibiotic Therapy (OPAT), which will need to cover increasingly resistant Gram-negative opportunists. We evaluated the activity of ertapenem/zidebactam, proposed for 2 + 2 g q24h administration.

**Materials and methods:**

MICs were determined, by BSAC agar dilution, for 1632 Enterobacterales submitted to the UK national reference laboratory for investigation of antimicrobial resistance.

**Results:**

Over 90% of *Escherichia coli* with AmpC, ESBLs, KPC, metallo- or OXA-48 carbapenemases were inhibited by ertapenem/zidebactam 1:1 at ertapenem’s current 0.5 mg/L breakpoint. For other major Enterobacterales, the proportions inhibited by ertapenem/zidebactam 1:1 at 0.5 mg/L were mostly 65% to 90% but were lower for *Klebsiella pneumoniae*/*oxytoca* with metallo- or OXA-48 β-lactamases. However, animal studies support an 8 mg/L breakpoint for ertapenem/zidebactam, based on a shortened *T*_>MIC_ being needed compared with ertapenem alone. On this basis ertapenem/zidebactam would count as active against 90%–100% of isolates in all groups except *K. pneumoniae*/*oxytoca* with MBLs (±OXA-48), where MICs and percent susceptibility vary substantially even with inocula within the BSAC acceptable range.

**Conclusions:**

Ertapenem/zidebactam has a proposed once-daily regimen well suited to OPAT. Even on highly conservative breakpoint projections, it has potential against MDR *E. coli*, including metallo-carbapenemase producers. If trial data sustain the 8 mg/L breakpoint indicated by animal experiments, its potential will extend widely across infections due to ESBL-, AmpC- and carbapenemase-producing Enterobacterales.

## Introduction

Once-daily antibiotic regimens are convenient and facilitate Outpatient Parenteral Antibiotic Therapy (OPAT). This mode of delivery seems set to expand, both because patients prefer to be treated at home and because COVID-19 will disrupt hospital medicine for several years to come.^1^

Among once-daily agents, teicoplanin and daptomycin are well suited to skin and skin-structure infections, being active against nearly all *Staphylococcus aureus* and streptococci.^2^ Dalbavancin and oritavancin have similar spectra and even simpler single-dose or once-weekly regimens.^2^ Ceftriaxone and aminoglycosides provide once-daily options with anti-Gram-negative coverage, but are constrained by resistance and, for aminoglycosides, toxicity.^3^ Global dissemination of uropathogenic *Escherichia coli* ST131 exerts a particular limitation; this widespread strain often combines ESBL production with resistance to aminoglycosides and fluoroquinolones.^4^ Ertapenem is a further once-daily option, covering ESBL-producing *E. coli*, including MDR ST131 isolates, but is limited by: (i) community spread of carbapenemase-producing Enterobacterales, particularly in south Asia and China^5^; (ii) low breakpoints; and (iii) being more vulnerable than other carbapenems to combinations of impermeability with ESBL or AmpC activity.^6^

A strategy to overcome these limitations is to increase the ertapenem dosage, and to add a triple-action diazabicyclooctane, aiming to: (i) support a higher breakpoint; (ii) inhibit carbapenemases; and (iii) achieve an enhancer effect by complementing ertapenem’s attack on PBP3 with concurrent targeting of PBP2. Ertapenem/zidebactam (WCK 6777) is being developed on this rationale, with a 2 + 2 g q24h regimen.^7^ We examined its activity against problem Enterobacterales, as submitted to the UK Health Security Agency’s (UKHSA) national reference laboratory.

## Materials and methods

### Bacteria and susceptibility testing

The test panel comprised around half of the Enterobacterales submitted to the UKHSA Antimicrobial Resistance and Healthcare-Associated Infections (AMRHAI) Reference Unit from July 2015 to July 2016. This collection, also including non-fermenters, was used for similar assessments of cefepime/tazobactam^8^ and cefepime/zidebactam,^9^ and comprises around half the set used for earlier assessments of ceftolozane/tazobactam^10^ and ceftazidime/avibactam.^11^ Most were referred owing to unusual resistance, particularly to carbapenems.

Species identification was by MALDI-TOF (Bruker Biotyper, Bremen, Germany). Susceptibility testing was by BSAC agar dilution on Iso-Sensitest agar^12^ (Oxoid, Basingstoke, UK), using a 1:1 gravimetric ratio of ertapenem:zidebactam, both from Wockhardt (Aurangabad, India). Susceptibility data for comparator antibiotics were published previously;^9^ a summary is provided in Table S1, available as Supplementary data at *JAC* Online. All MIC tests were performed in parallel, using the same inocula.

## Results and discussion

The interactions of zidebactam with partner β-lactams are complex and results should be interpreted with the following four points in mind.

First, ratio testing overcomes the problem that many Enterobacterales otherwise are inhibited by zidebactam at the low fixed concentrations (2–8 mg/L) conventionally used for β-lactamase inhibitors in MIC tests. Nonetheless, ratio MICs are inherently harder to interpret than when a straightforward β-lactamase inhibitor, lacking direct antibacterial activity, is incorporated at a fixed concentration.^13^

Secondly, breakpoints for ertapenem/zidebactam remain to be established. Values are low for unprotected ertapenem (EUCAST: S≤0.5, R > 0.5 mg/L; CLSI: S < 0.5, R > 1 mg/L) predicated upon a 1 g q24h regimen; however, ertapenem/zidebactam will be given at 2 g q24h, justifying a higher breakpoint. Moreover, recent humanized animal studies suggest that a shorter *T*_>MIC_ is needed than for ertapenem alone, with efficacy up to MICs of 8 mg/L.^7^

Thirdly, the AMRHAI Reference Unit receives a biased subset of isolates; AmpC and ESBL producers, in particular, are predominantly those with reduced susceptibility to carbapenems and (mistakenly) suspected of harbouring carbapenemases. Among the present 418 AmpC producers, 267 (63.9%) were non-susceptible to ertapenem (MIC > 0.5 mg/L), as were 43% (132/307) of the ESBL producers; by contrast, recent surveys show that unprotected ertapenem remains active against considerably larger proportions of unselected ESBL and AmpC producers.^14^

Last, in the case of MBL producers, MICs of zidebactam combinations vary according to whether they are determined with inocula at the high or low end of BSAC’s 1×10^4^ to 4 ×10^4^ cfu/spot acceptable range.^15^ The inoculum used here lies at the high end of this range, meaning that the proportions of MBL-producing isolates found resistant are maximal estimates.

### MICs by resistance group and prospective breakpoints

MIC distributions of ertapenem, zidebactam and ertapenem/zidebactam (1:1) are shown in Table 1 for all species combined and, wherever a mechanism group comprised over 100 isolates, also for its major component species, i.e. (i) *Escherichia coli*; (ii) *Klebsiella pneumoniae* and *Klebsiella oxytoca* pooled; and (iii) the pool of *Enterobacter* spp., *Citrobacter freundii* and *Klebsiella aerogenes*, which all have AmpC β-lactamases prone to mutational derepression.

**Table 1.** MIC distributions of ertapenem, zidebactam and their combination

Isolates (*n*)	Cumulative percent susceptible (at mg/L)												
	≤0.03	0.06	0.12	0.25	0.5	1	2	4	8	16	32	64	128
AmpC producers													
All (418)													
Ertapenem	9.1	12.2	16.3	20.1	36.1	51.0	70.1	84.4	91.9	95.2	97.6	99.0	100.0
Zidebactam	0.0	1.0	8.4	21.8	38.0	48.8	55.7	61.2	64.1	65.8	66.5	68.4	69.6
ERT-ZID 1:1	11.7	16.3	28.9	45.9	70.6	84.4	94.7	99.0	99.8	100.0	100.0	100.0	100.0
*E. coli* (47)													
Ertapenem	42.6	48.9	53.2	59.6	68.1	76.6	83.0	93.6	97.9	100.0	100.0	100.0	100.0
Zidebactam	0.0	6.4	31.9	48.9	72.3	83.0	87.2	91.5	91.5	91.5	91.5	93.6	95.7
ERT-ZID 1:1	46.8	53.2	70.2	83.0	91.5	97.9	100.0	100.0	100.0	100.0	100.0	100.0	100.0
*K. pneumoniae/oxytoca* (33)													
Ertapenem	24.2	30.3	39.4	42.4	57.6	75.8	81.8	84.8	90.9	97.0	97.0	100.0	100.0
Zidebactam	0.0	0.0	3.0	9.1	18.2	27.3	27.3	27.3	30.3	30.3	30.3	30.3	30.3
ERT-ZID 1:1	24.2	36.4	45.5	66.7	84.8	90.9	100.0	100.0	100.0	100.0	100.0	100.0	100.0
*Enterobacter/Citrobacter/K. aerogenes* (307)													
Ertapenem	2.0	3.6	6.2	9.8	27.7	44.3	68.1	84.0	91.5	94.5	97.4	98.7	100.0
Zidebactam	0.0	0.3	6.2	20.8	37.8	49.8	58.3	64.8	68.4	70.4	71.0	73.0	73.9
ERT-ZID 1:1	3.9	6.8	19.9	37.8	66.1	82.1	93.2	99.0	99.7	100.0	100.0	100.0	100.0
ESBL producers													
All (307)													
Ertapenem	16.0	25.7	34.5	42.0	57.0	69.1	80.5	86.6	93.2	96.4	99.7	100.0	100.0
Zidebactam	0.0	1.0	19.2	37.1	48.5	53.7	58.6	59.6	60.9	62.9	64.2	65.8	67.1
ERT-ZID 1:1	21.8	35.8	57.3	73.0	87.6	96.1	98.0	99.7	100.0	100.0	100.0	100.0	100.0
*E. coli* (145)													
Ertapenem	24.1	36.6	40.0	43.4	60.0	73.8	86.2	90.3	94.5	97.2	100.0	100.0	100.0
Zidebactam	0.0	2.1	37.2	64.1	77.9	85.5	91.0	91.7	92.4	93.8	93.8	94.5	94.5
ERT-ZID 1:1	32.4	42.8	69.7	80.0	92.4	98.6	99.3	100.0	100.0	100.0	100.0	100.0	100.0
*K. pneumoniae/oxytoca* (137)													
Ertapenem	6.6	14.6	29.2	42.3	58.4	69.3	78.1	83.2	92.0	96.4	99.3	100.0	100.0
Zidebactam	0.0	0.0	1.5	8.0	14.6	18.2	21.9	23.4	25.5	28.5	30.7	33.6	36.5
ERT-ZID 1:1	10.2	29.9	48.9	67.2	82.5	94.2	96.4	99.3	100.0	100.0	100.0	100.0	100.0
*Enterobacter/Citrobacter/K. aerogenes* (23)													
Ertapenem	17.4	21.7	30.4	30.4	30.4	34.8	56.5	82.6	91.3	91.3	100.0	100.0	100.0
Zidebactam	0.0	0.0	13.0	43.5	65.2	65.2	73.9	73.9	73.9	73.9	78.3	78.3	78.3
ERT-ZID 1:1	21.7	26.1	30.4	60.9	87.0	91.3	100.0	100.0	100.0	100.0	100.0	100.0	100.0
Enterobacterales producing KPC β-lactamases													
All (116)													
Ertapenem	1.7	2.6	2.6	2.6	2.6	3.4	6.9	18.1	27.6	38.8	75.9	89.7	96.6
Zidebactam	0.0	0.0	12.1	23.3	37.9	44.0	45.7	49.1	49.1	52.6	52.6	52.6	53.4
ERT-ZID 1:1	1.7	3.4	18.1	49.1	82.8	94.8	99.1	100.0	100.0	100.0	100.0	100.0	100.0
*E. coli* (20)													
Ertapenem	5.0	10.0	10.0	10.0	10.0	15.0	35.0	75.0	85.0	90.0	100.0	100.0	100.0
Zidebactam	0.0	0.0	65.0	75.0	80.0	95.0	95.0	100.0	100.0	100.0	100.0	100.0	100.0
ERT-ZID 1:1	5.0	15.0	70.0	90.0	95.0	100.0	100.0	100.0	100.0	100.0	100.0	100.0	100.0
*K. pneumoniae/oxytoca* (74)													
Ertapenem	1.4	1.4	1.4	1.4	1.4	1.4	1.4	1.4	8.1	17.6	68.9	86.5	94.6
Zidebactam	0.0	0.0	1.4	4.1	13.5	16.2	18.9	23.0	23.0	28.4	28.4	28.4	29.7
ERT-ZID 1:1	1.4	1.4	6.8	37.8	78.4	91.9	98.6	100.0	100.0	100.0	100.0	100.0	100.0
*Enterobacter/Citrobacter/K. aerogenes* (20)													
Ertapenem	0.0	0.0	0.0	0.0	0.0	0.0	0.0	25.0	45.0	70.0	85.0	95.0	100.0
Zidebactam	0.0	0.0	0.0	45.0	90.0	100.0	100.0	100.0	100.0	100.0	100.0	100.0	100.0
ERT-ZID 1:1	0.0	0.0	10.0	55.0	90.0	100.0	100.0	100.0	100.0	100.0	100.0	100.0	100.0
MBL-producing Enterobacterales													
All (210)													
Ertapenem	0.5	1.0	1.0	1.9	5.2	6.2	8.1	12.4	23.3	31.9	46.7	65.2	86.2
Zidebactam	0.0	0.5	21.4	35.7	44.8	48.1	50.0	51.4	52.4	54.3	56.2	57.1	58.1
ERT-ZID 1:1	1.0	1.9	23.8	38.6	54.8	62.4	71.9	78.1	84.8	91.4	95.2	98.6	99.5
*E. coli* (68)													
Ertapenem	1.5	1.5	1.5	1.5	4.4	4.4	4.4	5.9	8.8	14.7	30.9	44.1	83.8
Zidebactam	0.0	1.5	58.8	80.9	91.2	97.1	98.5	98.5	98.5	100.0	100.0	100.0	100.0
ERT-ZID 1:1	1.5	4.4	57.4	80.9	91.2	98.5	98.5	98.5	98.5	98.5	100.0	100.0	100.0
*K. pneumoniae/oxytoca* (106)													
Ertapenem	0.0	0.0	0.0	0.9	4.7	5.7	6.6	9.4	23.6	30.2	45.3	69.8	83.0
Zidebactam	0.0	0.0	0.0	2.8	7.5	10.4	13.2	16.0	17.9	20.8	24.5	25.5	27.4
ERT-ZID 1:1	0.0	0.0	0.0	3.8	12.4	27.6	34.3	48.6	61.0	73.3	84.8	91.4	98.1
*Enterobacter/Citrobacter/K. aerogenes* (30)													
Ertapenem	0.0	3.3	3.3	6.7	10.0	13.3	13.3	23.3	40.0	63.3	76.7	90.0	100.0
Zidebactam	0.0	0.0	16.7	56.7	80.0	80.0	80.0	80.0	80.0	80.0	80.0	83.3	83.3
ERT-ZID 1:1	3.3	3.3	23.3	43.3	70.0	76.7	90.0	90.0	93.3	100.0	100.0	100.0	100.0
Enterobacterales producing OXA-48 enzyme, ceftazidime S/I													
All (114)													
Ertapenem	0.9	1.8	1.8	3.5	14.9	23.7	53.5	75.4	87.7	90.4	95.6	97.4	98.2
Zidebactam	0.0	3.5	39.5	59.6	64.9	69.3	71.1	71.1	71.9	71.9	71.9	71.9	74.6
ERT-ZID 1:1	0.9	6.1	55.3	77.2	89.5	93.9	95.6	99.1	100.0	100.0	100.0	100.0	100.0
*E. coli* (60)													
Ertapenem	1.7	3.3	3.3	6.7	25.0	40.0	76.7	88.3	95.0	95.0	100.0	100.0	100.0
Zidebactam	0.0	6.7	65.0	81.7	90.0	95.0	96.7	96.7	96.7	96.7	96.7	96.7	98.3
ERT-ZID 1:1	1.7	11.7	88.3	98.3	98.3	98.3	100.0	100.0	100.0	100.0	100.0	100.0	100.0
*K. pneumoniae/oxytoca* (33)													
Ertapenem	0.0	0.0	0.0	0.0	6.1	6.1	27.3	66.7	81.8	84.8	87.9	90.9	93.9
Zidebactam	0.0	0.0	3.0	12.1	15.2	21.2	21.2	21.2	21.2	21.2	21.2	21.2	27.3
ERT-ZID 1:1	0.0	0.0	15.2	42.4	78.8	87.9	90.9	97.0	100.0	100.0	100.0	100.0	100.0
*Enterobacter/Citrobacter/K. aerogenes* (18)													
Ertapenem	0.0	0.0	0.0	0.0	0.0	5.6	27.8	50.0	77.8	88.9	94.4	100.0	100.0
Zidebactam	0.0	0.0	27.8	83.3	83.3	83.3	88.9	88.9	88.9	88.9	88.9	88.9	88.9
ERT-ZID 1:1	0.0	0.0	27.8	77.8	88.9	94.4	94.4	100.0	100.0	100.0	100.0	100.0	100.0
Enterobacterales producing OXA-48 enzyme, ceftazidime R													
All (136)													
Ertapenem	0.0	0.0	0.0	0.0	0.7	3.7	14.7	37.5	55.1	65.4	69.1	76.5	97.1
Zidebactam	0.0	1.5	12.5	31.6	36.0	39.7	46.3	50.0	52.9	54.4	54.4	56.6	60.3
ERT-ZID 1:1	0.0	0.7	14.0	41.2	60.3	70.6	83.8	94.9	100.0	100.0	100.0	100.0	100.0
*E. coli* (36)													
Ertapenem	0.0	0.0	0.0	0.0	2.8	11.1	33.3	58.3	77.8	97.2	97.2	97.2	100.0
Zidebactam	0.0	5.6	41.7	100.0	100.0	100.0	100.0	100.0	100.0	100.0	100.0	100.0	100.0
ERT-ZID 1:1	0.0	2.8	47.2	94.4	100.0	100.0	100.0	100.0	100.0	100.0	100.0	100.0	100.0
*K. pneumoniae/oxytoca* (77)													
Ertapenem	0.0	0.0	0.0	0.0	0.0	0.0	5.2	26.0	41.6	45.5	48.1	59.7	94.8
Zidebactam	0.0	0.0	0.0	1.3	3.9	7.8	15.6	22.1	26.0	28.6	28.6	29.9	36.4
ERT-ZID 1:1	0.0	0.0	0.0	15.6	41.6	54.5	72.7	90.9	100.0	100.0	100.0	100.0	100.0
*Enterobacter/Citrobacter/K. aerogenes* (21)													
Ertapenem	0.0	0.0	0.0	0.0	0.0	4.8	19.0	47.6	71.4	85.7	95.2	100.0	100.0
Zidebactam	0.0	0.0	9.5	28.6	47.6	57.1	61.9	61.9	66.7	66.7	66.7	76.2	76.2
ERT-ZID 1:1	0.0	0.0	9.5	47.6	66.7	81.0	95.2	100.0	100.0	100.0	100.0	100.0	100.0
*K. oxytoca* hyperproducing K1 enzyme (4)													
Ertapenem	25.0	75.0	75.0	75.0	75.0	75.0	100.0	100.0	100.0	100.0	100.0	100.0	100.0
Zidebactam	0.0	0.0	0.0	0.0	0.0	0.0	25.0	25.0	25.0	25.0	25.0	25.0	25.0
ERT-ZID 1:1	75.0	75.0	75.0	75.0	75.0	100.0	100.0	100.0	100.0	100.0	100.0	100.0	100.0
Enterobacterales producing GES carbapenemase (10)													
Ertapenem	0.0	0.0	0.0	0.0	0.0	10.0	40.0	40.0	50.0	60.0	70.0	70.0	70.0
Zidebactam	0.0	0.0	0.0	0.0	10.0	10.0	20.0	50.0	50.0	50.0	60.0	60.0	60.0
ERT-ZID 1:1	0.0	0.0	0.0	20.0	50.0	50.0	80.0	100.0	100.0	100.0	100.0	100.0	100.0
Enterobacterales producing other class A carbapenemase (9)													
Ertapenem	0.0	0.0	0.0	0.0	0.0	0.0	11.1	22.2	33.3	44.4	66.7	88.9	100.0
Zidebactam	0.0	0.0	22.2	44.4	55.6	55.6	55.6	55.6	55.6	55.6	55.6	66.7	66.7
ERT-ZID 1:1	0.0	0.0	44.4	55.6	66.7	66.7	77.8	88.9	100.0	100.0	100.0	100.0	100.0
Enterobacterales producing ESBL plus AmpC producers (27)													
Ertapenem	0.0	0.0	3.7	18.5	33.3	44.4	74.1	81.5	92.6	96.3	96.3	100.0	100.0
Zidebactam	0.0	3.7	11.1	33.3	55.6	81.5	81.5	81.5	81.5	81.5	81.5	81.5	81.5
ERT-ZID 1:1	3.7	7.4	29.6	51.9	74.1	85.2	100.0	100.0	100.0	100.0	100.0	100.0	100.0
Enterobacterales producing MBL (NDM) + OXA-48 enzymes (24)													
Ertapenem	0.0	0.0	0.0	0.0	0.0	0.0	0.0	0.0	0.0	0.0	0.0	4.2	12.5
Zidebactam	0.0	0.0	0.0	4.2	8.3	16.7	16.7	16.7	20.8	29.2	29.2	29.2	29.2
ERT-ZID 1:1	0.0	0.0	0.0	4.2	8.3	16.7	29.2	29.2	33.3	33.3	50.0	79.2	100.0
Impermeable (31)													
Ertapenem	12.9	19.4	25.8	35.5	41.9	58.1	83.9	93.5	93.5	100.0	100.0	100.0	100.0
Zidebactam	0.0	0.0	16.1	38.7	45.2	48.4	51.6	54.8	54.8	58.1	61.3	61.3	61.3
ERT-ZID 1:1	22.6	32.3	51.6	77.4	93.5	96.8	96.8	96.8	100.0	100.0	100.0	100.0	100.0
WT for β-lactamase (70)													
Ertapenem	71.4	80.0	81.4	82.9	84.3	95.7	95.7	97.1	98.6	100.0	100.0	100.0	100.0
Zidebactam	0.0	0.0	32.9	54.3	57.1	58.6	58.6	60.0	60.0	60.0	60.0	62.9	62.9
ERT-ZID 1:1	77.1	80.0	84.3	94.3	97.1	98.6	100.0	100.0	100.0	100.0	100.0	100.0	100.0
Unassigned mechanism(s), ceftazidime MIC ≤4 mg/L (58)													
Ertapenem	39.7	43.1	53.4	60.3	70.7	75.9	86.2	96.6	100.0	100.0	100.0	100.0	100.0
Zidebactam	0.0	1.7	29.3	44.8	46.6	48.3	53.4	56.9	58.6	60.3	67.2	70.7	72.4
ERT-ZID 1:1	43.1	51.7	65.5	79.3	93.1	98.3	100.0	100.0	100.0	100.0	100.0	100.0	100.0
Unassigned mechanism(s), ceftazidime MIC 8–32 mg/L (20)													
Ertapenem	0.0	10.0	25.0	30.0	45.0	55.0	65.0	70.0	80.0	85.0	90.0	100.0	100.0
Zidebactam	0.0	0.0	0.0	5.0	15.0	15.0	20.0	20.0	20.0	25.0	25.0	35.0	45.0
ERT-ZID 1:1	10.0	25.0	30.0	50.0	65.0	75.0	85.0	100.0	100.0	100.0	100.0	100.0	100.0
Unassigned mechanism(s), ceftazidime MIC >32 mg/L (64)													
Ertapenem	9.4	10.9	15.6	17.2	18.8	20.3	31.3	37.5	45.3	54.7	76.6	92.2	96.9
Zidebactam	0.0	3.1	4.7	9.4	10.9	14.1	18.8	21.9	25.0	31.3	31.3	35.9	35.9
ERT-ZID 1:1	10.9	14.1	17.2	21.9	31.3	50.0	68.8	89.1	98.4	98.4	100.0	100.0	100.0
*K. pneumoniae* type 1 unknown (14)^a^													
Ertapenem	7.1	14.3	21.4	28.6	35.7	50.0	57.1	85.7	85.7	85.7	92.9	100.0	100.0
Zidebactam	0.0	0.0	14.3	14.3	14.3	14.3	21.4	21.4	21.4	28.6	28.6	28.6	28.6
ERT-ZID 1:1	14.3	14.3	35.7	50.0	64.3	85.7	92.9	100.0	100.0	100.0	100.0	100.0	100.0

ERT-ZID, ertapenem/zidebactam; S, susceptible; I, intermediate/increased dose susceptible; R resistant. ^a^See Livermore *et al*.^11^ for a description of this group and its phenotype.

Over 90% of *E. coli* with AmpC, ESBLs, KPC, MBLs and OXA-48 carbapenemases were inhibited by ertapenem/zidebactam at ertapenem’s 0.5 mg/L breakpoint, whereas ertapenem alone inhibited only 60.0% to 68.1% of the ESBL- and AmpC-producing *E. coli* and 2.8% to 25% of carbapenemase-producing *E. coli.* This gain substantially reflected the inherent antibacterial activity of zidebactam. Nonetheless, zidebactam 0.5 mg/L alone inhibited fewer *E. coli* isolates in most categories than ertapenem/zidebactam; exceptions were MBL producers (91.2% inhibited by both zidebactam alone and ertapenem/zidebactam) and ceftazidime-resistant OXA-48 β-lactamase producers (100% inhibited by both zidebactam alone and ertapenem/zidebactam). At 8 mg/L, ertapenem/zidebactam inhibited all *E. coli* tested, except for 1/68 MBL producers.

For other species besides *E. coli*, the proportions of isolates in each resistance mechanism group inhibited by ertapenem/zidebactam 0.5 mg/L were mostly between 65% and 90%, exceeding the proportions inhibited by ertapenem or zidebactam 0.5 mg/L alone. Lower proportions were seen for: (i) MBL-producing *K. pneumoniae*/*oxytoca* (12.4% inhibited); (ii) ceftazidime-resistant *K. pneumoniae*/*oxytoca* with OXA-48-like enzymes (41.6%); (iii) Enterobacterales (23/24 *Klebsiella* spp.) with both MBLs and OXA-48-like enzymes (8.3%); and (iv) highly ceftazidime-resistant isolates with undetermined mechanisms (31.3%). If, however, future clinical trial data support the 8 mg/L breakpoint indicated by the animal studies of Gethers *et al.*,^7^ ertapenem/zidebactam would count as active against 90%–100% of isolates in all species/mechanism groups except (i) MBL-producing *K. pneumoniae*/*oxytoca* (61.0% inhibited); and (ii) isolates with both MBL and OXA-48 carbapenemases (33.3% inhibited). It should be restressed that the proportions inhibited in the latter groups would be expected to rise with inocula at the lower end of the acceptable inoculum range, rather than the higher end, as used.^15^ For illustration, when 33 Enterobacterales with MBLs (half also with OXA-48) previously found to be resistant to cefepime/zidebactam 8 + 8 mg/L were tested with ertapenem/zidebactam, just 9/33 were inhibited at 8 + 8 mg/L with the inocula (*c*. 3×10^4^ to 6×10^4^ cfu/spot) used here, but 23/33 were inhibited with inocula at the lower end of the BSAC’s acceptable range (*c*. 1×10^4^ to 2×10^4^ cfu/spot).^15^

### Performance of ertapenem/zidebactam against isolates highly resistant to both components

Table 2 shows the distribution of ertapenem/zidebactam MICs for Enterobacterales resistant to both zidebactam and ertapenem alone at 32 mg/L. Despite high-level resistance to both its components, MICs of ertapenem/zidebactam were in the range 2–8 mg/L for many of these isolates. For those with KPC, ESBLs and AmpC enzymes, gain of activity reflects β-lactamase inhibition. However, this cannot be the case for isolates with enzymes not inhibited by zidebactam, notably OXA-48-like or metallo types; here, regain reflects the enhancer effect.

**Table 2.** MICs of ertapenem/zidebactam 1:1 for Enterobacterales isolates with MICs ≥32 mg/L for each agent alone

Isolates (*n*)	No. of isolates with indicated MIC (mg/L)										
	0.25	0.5	1	2	4	8	16	32	64	128	>128
AmpC hyperproducers (14)				4	8	2					
ESBL producers (5)			1	1	2	1					
ESBL + AmpC (1)				1							
KPC carbapenemases (47)	11	22	9	4	1						
GES carbapenemases (1)				1							
Other class A carbapenemases (4)		1		1	1	1					
MBL (62)	1	3		8	9	11	13	7	7	2	1
MBL (NDM) + OXA-48 (17)			1	2		1		2	6	5	
OXA-48 ceftazidime S/I (7)			1	2	3	1					
OXA-48 ceftazidime R (32)			4	9	12	7					
Unassigned mechanism(s), ceftazidime MICs 8–32 mg/L (3)				1	2						
Unassigned mechanism(s), ceftazidime MICs >32 mg/L (21)			1	6	9	4		1			
*K. pneumoniae* type I unknown^a^ (2)				1	1						

S, susceptible; I, intermediate/increased dose susceptible; R resistant. ^a^See Livermore *et al.*^11^ for a description of this group and its phenotype.

Strikingly, barring a single isolate with an unassigned mechanism, resistance to ertapenem/zidebactam 8 mg/L was seen *only* among MBL producers and those with both MBLs and OXA-48-like enzymes.

### Conclusions

Addition of zidebactam extends the activity of ertapenem to include many carbapenemase producers as well as isolates with combinations of impermeability and ESBL or AmpC activity. This is important, given (i) the accumulation of pathogens with these mechanisms; and (ii) in India, China and parts of Europe, the diffusion of carbapenemase-producing Enterobacterales into the community.^5,16^

The potential of the combination will depend crucially on what breakpoints can be supported. With a low breakpoint (0.5–1 mg/L), utility against MDR strains will largely relate to *E. coli*, which is responsible for around 80% of UTIs, including complicated and ascending cases. If, however, a breakpoint of 8 mg/L is justified, utility will extend far more widely, encompassing almost all combinations of major Enterobacterales species and prevalent resistance mechanisms.

In either case, the scope for deployment as OPAT is crucial, differentiating ertapenem/zidebactam from cefiderocol and various other developmental combinations, notably cefepime/zidebactam, cefepime/taniborbactam and aztreonam/avibactam. These have similarly broad activity against ESBL-, AmpC- and carbapenemase-producing Enterobacterales but require q8h regimens.

The potential for OPAT use is of particular importance, given COVID-19’s continuing disruption of secondary care. This is especially marked in countries, e.g. the UK, where hospitals ordinarily function in a high-throughput, low-capacity model.^17^ Whilst vaccination reduces severe illness, mass vaccination has failed to terminate the COVID-19 pandemic, and infection remains highly prevalent in countries with high vaccine coverage. Ultimately, it is to be anticipated that SARS-CoV-2 will become as endemic and benign as the four common cold coronaviruses. But, during the years required for this balance to stabilize, the virus will continue to engender disruption, causing nosocomial outbreaks and hospital staff absences. Simultaneously, there is a large and growing backlog of patients awaiting elective procedures or with undiagnosed illness, including cancers.^18^ Once finally admitted, these patients will be older, sicker and more prone to infections by MDR opportunist bacteria than if their care had not been disrupted by the pandemic.

Partial answers to this nexus of unfolding challenges include alleviating pressures within hospitals by treating more patients in the community. In the case of antibiotics, this will drive the use of OPAT, which will increasingly need to cover MDR pathogens. These shifts are creating the niche for ertapenem/zidebactam. Its ultimate utility—as an anti-*E. coli* or broader agent—will depend greatly on the breakpoints assigned.

## Supplementary Material

dkac280_Supplementary_Data

## Transparency declarations

D.M.L.: Advisory Boards or *ad hoc* consultancy—Accelerate, Antabio, Centauri, Genpax, Meiji, Menarini, Mutabilis, Nordic, Paion, Paragraf, ParaPharm, Pfizer, QPEX, Shionogi, Sumitovant, Summit, T.A.Z., Thermo Fisher, Venatorx, Wockhardt and Zambon; paid lectures—bioMérieux, GSK, Hikma, Merck/MSD, Menarini, Nordic, Pfizer and Shionogi; relevant shareholdings or options—Dechra, Genpax, GSK, Merck and Perkin-Elmer, amounting to less than 10% of portfolio value. He also has nominated holdings in Arecor, Avacta, Diaceutics, Creo Medical, Evgen, Genedrive, Poolbeg, Renalytics AI and Trellus (all with research/products pertinent to medicines or diagnostics) through Enterprise Investment Schemes but has no authority to trade these shares directly. All other authors are employees of the UKHSA’s Antimicrobial Resistance and Healthcare Associated Infections Reference Unit, which has received financial support for conference attendance, lectures, research projects, or contracted evaluations from numerous sources, including Accelerate Diagnostics, Achaogen Inc., Allecra Therapeutics, Amplex, AstraZeneca UK Ltd, AusDiagnostics, Basilea Pharmaceutica, Becton Dickinson Diagnostics, bioMérieux, Bio-Rad Laboratories, BSAC, Cepheid, Check-Points B.V., Cubist Pharmaceuticals, Department of Health, Enigma Diagnostics, Food Standards Agency, GlaxoSmithKline Services Ltd, Helperby Therapeutics, Henry Stewart Talks, IHMA Ltd, Innovate UK, Integra holdings, Kalidex Pharmaceuticals, Melinta Therapeutics, Merck Sharpe & Dohme Corp., Meiji Seika Pharma Co. Ltd, Mobidiag, Momentum Biosciences Ltd, Neem Biotech, Nordic Pharma Ltd, Norgine Pharmaceuticals, Paratek Pharmaceuticals, Rempex Pharmaceuticals Ltd, Roche, Rokitan Ltd, Smith & Nephew UK Ltd, Shionogi & Co. Ltd, Trius Therapeutics, T.A.Z., Venatorx Pharmaceuticals and Wockhardt Ltd.

## Supplementary data

Table S1 is available as Supplementary data at *JAC* Online.
