# Supplementary material for: Activity of ertapenem/zidebactam (WCK 6777) against problem Enterobacterales
Source: J Antimicrob Chemother. 2022 Aug 16;77(10):2772–8. doi: 10.1093/jac/dkac280 (PMC9384802; doi:10.1093/jac/dkac280)
Supplement: dkac280_Supplementary_Data [file dkac280_supplementary_data.docx]

**Supplementary data**

**Table S1.** Susceptibility of the strain panel to comparator antibiotics

|  | Cefepime | | CAZ/AVI | TOL/TAZ | PIP/TAZ | Meropenem | |
| --- | --- | --- | --- | --- | --- | --- | --- |
| Enterobacterales  Breakpoints mg/L (EUCAST S and S+I) | <1 | <4 | <8+4 | <2+4 | <8+4 | <2 | <8 |
| AmpC (n=418) | 66.8% | 91.1% | 99.8% | 44.5% | 28.7% | 94.0% | 98.6% |
| *E. coli* (47) | 70.2% | 82.3% | 100% | 66.0% | 57.4% | 97.9% | 100% |
| *K. pneumoniae/ K. oxytoca* (33) | 100% | 87.9% | 100% | 72.7% | 30.3% | 97.0% | 100% |
| *Enterobacter/Citrobacter/ K. aerogenes* (307) | 63.8% | 90.9% | 99.7% | 109 | 35.5% | 93.2% | 98.0% |
| ESBL (n=307) | 12.8% | 22.2% | 100% | 64.7% | 41.2% | 95.8% | 99.7% |
| *E. coli* (145) | 9.7% | 20.7% | 100% | 72.4% | 51.0% | 96.6% | 100% |
| *K. pneumoniae/ K. oxytoca* (137) | 12.4% | 18.2% | 100% | 59.1% | 31.4% | 94.9% | 99.4% |
| *Enterobacter/Citrobacter/ K. aerogenes* (23) | 34.1% | 52.2% | 100% | 47.8% | 34.8% | 95.7% | 100% |
| KPC carbapenemases (n=116) | 3.5% | 52.6% | 99.1% | 6.9% | 2.6% | 18.1% | 60.3% |
| *E. coli* (20) | 15% | 55% | 100% | 25% | 10% | 75% | 95% |
| *K. pneumoniae/ K. oxytoca* (74) | 4.1% | 56.8% | 98.6% | 4.1% | 1.4% | 2.8% | 50% |
| *Enterobacter/Citrobacter/ K. aerogenes* (20) | 0% | 35% | 100% | 0% | 0% | 20% | 70% |
| MBL carbapenemases (n=210) | 1.4% | 3.8% | 4.3% | 1.4% | 2.0% | 10.5% | 28.6% |
| *E. coli* (68) | 1.5% | 4.5% | 3.0% | 1.5% | 1.5% | 4.5% | 21.0% |
| *K. pneumoniae/ K. oxytoca* (106) | 0% | 2.8% | 4.7% | 0% | 0.9% | 12.3% | 25.5% |
| *Enterobacter/Citrobacter/ K. aerogenes* (30) | 6.7% | 6.7% | 6.7% | 3.3% | 13.3% | 20.0% | 43.3% |
| OXA-48 ceftazidime-S/I (n=114) | 79.0% | 95.6% | 100% | 90.4% | 0.9% | 84.8% | (96.5%) |
| *E. coli* (60) | 78.3% | 98.3% | 100% | 95.0% | 1.7% | 96.6% | 100% |
| *K. pneumoniae/ K. oxytoca* (33) | 78.8% | 90.9% | 100% | 84.8% | 0% | 75.8% | 87.9% |
| *Enterobacter/Citrobacter/ K. aerogenes* (18) | 77.8% | 94.4% | 100% | 83.3% | 0% | 77.8% | 100% |
| OXA-48-ceftazidime-R (n=136) | 9.6% | 19.1% | 98.5% | 6.6% | 0% | 62.5% | (74.3%) |
| *E. coli* (36) | 2.8% | 13.9% | 97.2% | 11.1% | 5.6% | 97.2% | 97.2% |
| *K. pneumoniae/ K. oxytoca* (77) | 5.2% | 11.7% | 98.7% | 2.6% | 0% | 45.5% | 57.1% |
| *Enterobacter/Citrobacter/ K. aerogenes* (21) | 38.1% | 57.1% | 100% | 14.3% | 0% | 71.4% | 95.2% |
| K1 hyperproducing *K. oxytoca* (n=4) | 25.0% | 75.0% | 100% | 75.0% | 0% | 100% | 100% |
| GES carbapenemases (n=10) ^a^ | 20.0% | 30.0% | 100% | 0% | 0% | 40.0% | 60.0% |
| Other (i.e. not GES or KPC) class A carbapenemase (n=9) ^b^ | 100% | 100% | 100% | 100% | 88.9% | 11.1% | 44.4% |
| ESBL + AmpC (n=27) ^c^ | 14.8% | 25.9% | 100% | 18.5% | 14.8% | 100% | 100% |
| MBL (NDM) + OXA-48 (n=24) ^d^ | 0% | 0% | 4.2% | 0% | 0% | 0% | 4.2% |
| Impermeability (n=31) ^e^ | 74.2% | 93.6% | 100% | 90.3% | 48.4% | 96.8% | 100% |
| Wildtype for β-lactamase (n=69) ^f^ | 98.6% | 100% | 100% | 98.6% | 94.9% | 100% | 100% |
| Unassigned ceftazidime MIC <4 mg/L (n=58) ^g^ | 70.7% | 86.2% | 100% | 93.1% | 48.3% | 98.3% | 100% |
| Unassigned ceftazidime MIC 8-32 mg/L (n=20) ^h^ | 40.0% | 60.0% | 100% | 50.0% | 30.0% | 75.0% | 90.0% |
| Unassigned ceftazidime MIC >32 mg/L (n=64) ^i^ | 9.4% | 12.5% | 96.88% | 25.0% | 15.6% | 56.3% | 89.1% |
| *K. pneumoniae* ‘Type 1 unknown’ (n=14) | 28.6% | 78.6% | 100% | 57.1% | 14.3% | 85.7% | 100% |

**Table S1** - notes

^a^ *E. coli* (n=4), *Klebsiella* spp. (n=4), *Enterobacter* spp. (n=0), Others (n=2)

^b^ *E. coli* (n=0), *Klebsiella* spp. (n=0), *Enterobacter* spp. (n=6), Others (n=3)

^c^ *E. coli* (n=11), *Klebsiella* spp. (n=3), *Enterobacter* spp. (n=12), Others (n=1)

^d^ *E. coli* (n=1), *Klebsiella* spp. (n=23), *Enterobacter* spp. (n=0), Others (n=0)

^e^ *E. coli* (n=12), *Klebsiella* spp. (n=17), *Enterobacter* spp. (n=3), Others (n=0)

^f^ *E. coli* (n=15), *Klebsiella* spp. (n=14), *Enterobacter* spp. (n=17), Others (n=24)

^g^ *E. coli* (n=29), *Klebsiella* spp. (n=21), *Enterobacter* spp. (n=3), Others (n=5)

^h^ *E. coli* (n=2), *Klebsiella* spp. (n=16), *Enterobacter* spp. (n=2), Others (n=0)

^i^ *E. coli* (n=11), *Klebsiella* spp. (n=53), *Enterobacter* spp. (n=0), Others (n=0)

Abbreviations: S, susceptible; I, EUCAST increased-dose susceptible; R resistant; CAZ/AVI, ceftazidime/avibactam; TOL/TAZ, ceftolozane/tazobactam, PIP/TAZ, piperacillin/tazobactam.
